# Two new species of *Endocarpon* (Verrucariaceae, Ascomycota) from China

**DOI:** 10.1038/s41598-017-07778-5

**Published:** 2017-08-03

**Authors:** Tao Zhang, Meng Liu, Yan-Yan Wang, Zhi-Jun Wang, Xin-Li Wei, Jiang-Chun Wei

**Affiliations:** 1 State Key Laboratory of Mycology, Institute of Microbiology, Chinese Academy of Sciences, Beijing, 100101 PR China; 2 The College of Life Science, Southwest Forestry University, Kunming, 650224 PR China; 3 University of Chinese Academy of Sciences, Beijing, 100049 PR China

## Abstract

*Endocarpon* species are key components of biological soil crusts. Phenotypic and systematic molecular analyses were carried out to identify samples of *Endocarpon* collected from the southeast edge of the Tengger Desert in China. These morphological and molecular analyses revealed two previously undescribed species that form highly supported independent monophyletic clades within *Endocarpon*. The new taxa were named *Endocarpon deserticola* sp. nov. and *E. unifoliatum* sp. nov. Furthermore, our results indicated that the newly developed protein coding markers adenylate kinase (ADK) and ubiquitin-conjugating enzyme h (UCEH) are useful for assessing species boundaries in phylogenic analyses.

## Introduction

Biological soil crusts (BSCs) are intimate association between soil particles and biological communities composed of mosses, lichens, cyanobacteria and heterotrophs living at the soil surface^1, 2^. Soil particles are aggregated through the presence and activity of the biota mentioned above, and the resultant living crusts cover more than 40% of the Earth’s terrestrial surface as a coherent layer^1, 2^. BSCs play an important role in carbon and nitrogen fixation and soil stabilization of desert ecosystems^2–4^. According to the existence of different dominant species during the development of BSCs, it could be mainly divided into algae crust, lichen crust and moss crust^5^, among which the lichen crust is more compact and has stronger ability in carbon and nitrogen fixation^6^.

**Figure 1:**
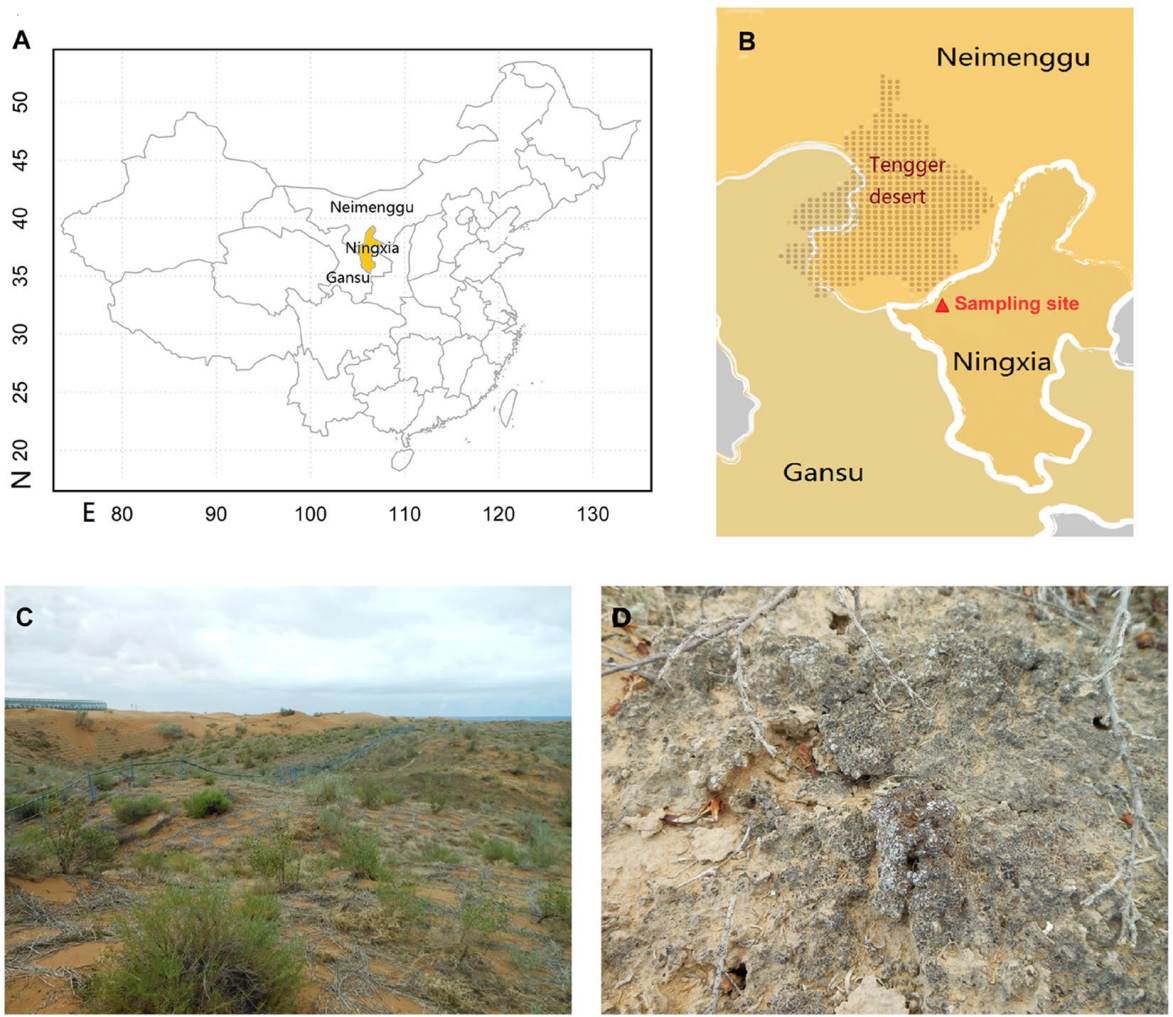
The location of sampling site and field overview. (**A**) The location of the sampling site in China, highlighted with orange color (created using R3.4.0); (**B**) Partial magnification of the detailed sampling site marked by a solid red triangle, situated in Ningxia Hui Autonomous Region and the southeast edge of the Tengger Desert (created using the drawing tool software Microsoft Paint (Windows 8.0); (**C**) Field overview of the sampling site; (**D**) Detailed view of *Endocarpon* spp. in the BSC.

The lichen-forming fungal genus *Endocarpon* Hedw. is a key component of BSCs in the arid and semiarid regions, which can aggregate soil particles using squamulose thallus or rhizines at their lower surface^1^. *Endocarpon* crusts play important ecological roles in desert ecosystems, such as stabilising sand dunes^7^, preventing soil wind erosion^8^, and promoting soil fertility^4^. In China, this genus has been reported as dominant group growing in Shapotou Region^9^, where is the southeast edge of the Tengger Desert (Fig. 1). As dominant species, *Endocarpon pusillum* Hedw. has been intensively studied from many aspects, such as physiology under desiccation and starvation stress^10^, photosynthetic rate^11^, genome^12^, transcriptome^13^ and stress resistance functional protein^14^. And all the above results supported that *Endocarpon pusillum* has good drought resistant ability, which could be one potential species used in the prospective ‘desert biocarpet engieering’ (DBCE)^15^, which has been proposed by Wei^16^, referring to developing during a short period into a dominant protection system instead of the naked desert with the help of the artificial inoculation of the microorganisms.

The genus *Endocarpon* belongs to the Verrucariaceae, Verrucariales, Chaetothyriomycetidae, Eurotiomycetes, and Ascomycota^17^ and is characterised by a squamulose thallus, immersed perithecioid ascomata with hymenial algae, and asci with (1-)2(-8) muriform ascospores. The key characteristics delimiting *Endocarpon* species generally include squamule morphology and colour, presence or absence and colour of rhizines, amounts of perithecia and ascospores, and ascospore size. Although molecular data have greatly strengthened our understanding of the evolution and phylogenetic relationships of lichen-forming fungi, resulting in dramatic changes in their classification over the past few decades^18–24^, the phylogenetic relationships in *Endocarpon* are still not well understood. Until now, only a few studies have provided insight into the molecular phylogeny of a limited number of *Endocarpon* species, including *E. pusillum*, *E. adscendens* (Anzi) Müll. Arg., *E. crystallinu* J.C. Wei & Jun Yang*, E. tenuissimum* (Degel.) Lendemer & E.A. Tripp, *E. psorodeum* (Nyl.) Th. Fr., *E. petrolepideum* (Nyl.) Nyl. and *E. pallidulum* Ach^9, 25–30^. Globally, the genus comprises approximately 50 species^31^ based on studies conducted in China^15, 16, 32–36^ and other regions of the world^37–45^.

In order to seek for new species resource with sand-stabilisation potential besides *Endocarpon pusillum*, we carried out taxonomic study on Chinese *Endocarpon* and two specific species drew our attention because they were not assignable to any known species based on phenotypic characteristics. In view of insufficiency of phylogenetic data in the genus *Endocarpon*, we aim to study the two species based on both phenotypic traits and phylogeny and accumulate more DNA data for the further study.

Nowadays, besides nrDNA ITS region, which is often used in fungal species delimitation^46^, some protein-coding genes (e.g., RPB1, MCM7) have also been successfully used^47^. In this study, two protein-coding genes (ADK and UCEH) were developed and used for the first time for phylogenetic analyses. ADK is a phosphotransferase enzyme that catalyses the interconversion of adenine nucleotides, thus playing an important role in cellular energy homeostasis^48^. UCEH is a subunit of ubiquitin-conjugating enzymes and performs the second step of the ubiquitination reaction that targets a protein for degradation by the proteasome^49^. We newly designed the primers for ADK and UCEH based on the cDNA library of *E. pusillum* ^50^ in this study. Our major goals are (1) to describe the phenotypic characteristics of these two species, (2) understand the phylogenetic relationships in the genus *Endocarpon* and (3) as well as develop two new protein coding markers (ADK, UCEH) to strengthen the phylogenetic information.

## Results

### Molecular phylogeny

The aligned matrix contained 476 unambiguous nucleotide position characters for the internal transcribed spacer (ITS), 679 for the adenylate kinase (ADK), and 279 for the ubiquitin-conjugating enzyme h (UCEH). The final alignment of the concatenated data set was 1434 positions in length. Seventy-six sequences were newly generated for this study (Table 1).

**Table 1:** Specimen information and GenBank accession numbers for the taxa used in this study.

No.	Species name	Collector, Coll. no. & time	Locality	GenBank no. of ITS	GenBank no. of UCEH	GenBank no. of ADK
1	*E. adsurgens*	T. Zhang & S.N. Cao, SPT3–3, Aug.31, 2008	Ningxia, China	KX538743	KX538767	KX538710
2	*E. adsurgens*	J. Yang & M.R. Huang, QH014, Sep.10, 2005	Qinghai, China	KX538742	KX538766	KX538712
3	*E. adsurgens*	J. Yang & M.R. Huang, GS158, May 17, 2005	Gansu, China	KX538741	KX538765	KX538711
4	*E. adsurgens*	DQ12066	Yunnan, China	—	—	KX538731
5	*E. adsurgens*	DQ12064	Yunnan, China	—	—	KX538730
6	*E. adsurgens*	Q.M. Zhou *et al*., HL12078, Sep.16, 2012	Ningxia, China	—	—	KX538725
7	*E. adsurgens*	Q.M. Zhou *et al*., HL12073, Sep.16, 2012	Ningxia, China	—	—	KX538733
8	*E. adsurgens*	Q.M. Zhou *et al*., HL12071, Sep.16, 2012	Ningxia, China	—	—	KX538732
9	*E. crystallinum*	T. Zhang, Z07083, Jan.12, 2007	Ningxia, China	KX538746	KX538770	KX538715
10	*E. crystallinum*	J. Yang & T. Zhang, SPT363, Aug.25, 2006	Ningxia, China	KX538744	KX538768	KX538713
11	*E.crystallinum*	T. Zhang, Z07018, Jan.12, 2007	Ningxia, China	KX538745	KX538769	KX538714
12	*E. deserticola*	T. Zhang & S.N. Cao, SPT3–10, Aug.31, 2008	Ningxia, China	KX538748	KX538771	KX538716
13	*E. deserticola*	T. Zhang & J. Yang, SPT295, Apr.18, 2007	Ningxia, China	—	—	KX538726
14	*E. deserticola*	T. Zhang, SPT10078, Apr.10, 2010	Ningxia, China	KX538749	KX538772	KX538717
15	*E. deserticola*	T. Zhang, Z10010, Apr.8, 2010	Ningxia, China	KX538750	KX538773	KX538718
16	*E. deserticola*	T. Zhang, Z07090, Jan.12, 2007	Ningxia, China	KX538747	—	—
17	*E. nigromarginatum*	J. Yang & T. Zhang SPT 191, Apr.17, 2007	Ningxia, China	KX538751	—	—
18	*E. nigromarginatum*	DQ12003	Yunnan, China	—	—	KX538727
19	*E. nigromarginatum*	J. Yang & T. Zhang, SPT268, Apr.13, 2007	Ningxia, China	KX538752	KX538774	—
20	*E. pusillum*	J. Yang & T. Zhang, SPT294, Apr.18, 2007	Ningxia, China	KX538754	KX538776	KX538720
21	*E. pusillum*	J. Yang & T. Zhang, SPT190, Apr.17, 2007	Ningxia, China	KX538753	KX538775	KX538719
22	*E. pusillum*	Q.M. Zhou *et al*., HL12084, Sep.16, 2012	Ningxia, China	—	—	KX538736
23	*E. pusillum*	K. Chen *et al*., HL12213, Sep.16, 2012	Ningxia, China	—	—	KX538737
24	*E. pusillum*	Q.M. Zhou *et al*., HL12051, Sep.16, 2012	Ningxia, China	—	—	KX538735
25	*E. pusillum*	K. Chen *et al*., HL12029, Sep.16, 2012	Ningxia, China	—	—	KX538734
26	*E. pusillum*	Q.M. Zhou *et al*., HL12227, Sep.16, 2012	Ningxia, China	—	—	KX538738
27	*E. sinense*	J. Yang & E.R. Zhang, GS034, Oct.27, 2004	Gansu, China	KX538757	KX538779	KX538723
28	*E. sinense*	J. Yang & E.R. Zhang, GS031, Oct.27, 2004	Gansu, China	KX538756	KX538778	KX538722
29	*E. sinense*	J. Yang & E.R. Zhang, GS030, Oct.27, 2004	Gansu, China	KX538755	KX538777	KX538721
30	*E. unifoliatum*	T. Zhang, SPT10063, Apr.9, 2010	Ningxia, China	KX538761	KX538782	KX538724
31	*E. unifoliatum*	T. Zhang, SPT10062, Apr.9, 2010	Ningxia, China	KX538760	—	KX538729
32	*E. unifoliatum*	T. Zhang, SPT10047, Apr.9, 2010	Ningxia, China	KX538759	KX538781	KX538728
33	*E. unifoliatum*	J. Yang & T. Zhang, SPT187, Apr.19, 2007	Ningxia, China	KX538758	KX538780	KX538739
34	*E. unifoliatum*	T. Zhang, Z10020, Apr.8, 2010	Ningxia, China	KX538762	—	—
35	*Dermatocarpon miniatum*	L.Y. Sun, S707, Aug.2, 2007	Jilin, China	KX538764	KX538784	—
36	*D. miniatum*	L.Li et al.,WLS072, Aug.27, 2009	Hebei, China	KX538763	KX538783	KX538740
37*	*D. dolomiticum*	Harris 25421	Missouri, USA	EF014211	—	—
38*	*D. miniatum*	Buck 47331	Wales, England	EF014192	—	—
39*	*D. miniatum*	Y. Zhang et al. A33	China	JQ740012	—	—
40*	*D. muehlenbergii* var. *tenue*	Heiðmarsson 1137	Arizona, USA	AF333128	—	—
41*	*E. pallidulum*	0047525 (DUKE)	North Carolina, USA	DQ826735	—	—
42*	*E. petrolepideum*	U-492F (DUKE)	Maryland, USA	KF959778	—	—
43*	*E. psorodeum*	CG 684 (DUKE)	Estonia	KF959779	—	—
44*	*E. adscendens*	CG 671 (DUKE)	Switzerland	KF959777	—	—
45*	*E. pusillum*	CG 470 (DUKE)		JQ927447	—	—
46*	*E. tenuissimum*	Lendemer 27013, 2010	North Carolina, USA	KM371593	—	—
47*	*E. tenuissimum*	Lendemer 29447, 2010	North Carolina, USA	KM371592	—	—
48*	*Staurothele areolata*	AFTOL-ID 2291, C. Gueidan 378 (MARSSJ)		EU006543	—	—
49*	*S. areolata*	CG378		JQ927448	—	—
50*	*S. fissa*	Orange 16265	United Kingdom	FJ645265	—	—
51*	*S. frustulenta*	AFTOL-ID 697		DQ826736	—	—
52*	*S. frustulenta*	long15	United States	KC990385	—	—
53*	*S. fuscocuprea*	SS087, S. Savic 3091 (UPS)	Sweden	EU553513	—	—
54*	*S. rupifraga*	SS001, S. Savic 3003 (UPS)	Sweden	EU553490	—	—
55*	*Verrucaria devensis*	Orange 21331	Wales, England	KF819519	—	—
56*	*V. hydrophila*	Orange 20829	Wales, England	JX848580	—	—
57*	*V. placida*	Orange 17212	Norway	JX848573	—	—
58*	*V. placida*	Orange 19380	Germany	JX848574	—	—
59*	*V. rosula*	Orange 20542	Wales, England	JX848577	—	—
60*	*Willeya diffractella*	Lendemer 28379		KM371614		
61*	*W. fusca*	BM CG1877		NR136067		
62*	*W. laevigata*	BM CG1852		NR136068		
63*	*W. pallidopora*	CG1926		KF959791		
64*	*W. protrudens*	BM CG1945		NR136066		

*DNA sequences were downloaded from GenBank. Othe specimens were sequenced by the authors; all sequences were deposited in HMAS-L; missing sequences are indicated by dashes.

The single-locus gene trees for the three markers are illustrated in Figures S1–S3. The topologies of the single-locus phylogenies did not exhibit obviously supported conflicts (i.e. bootstrap values ≥75%), and thus they were analysed in a concatenated data matrix. The best-fitting models corresponding to the three single-locus markers are listed in Table 2.

**Table 2:** The best-fitting models corresponding to the three single-locus genes used in the phylogenetic analyses.

Gene name	Best-fitting model	AIC	−lnL
ITS	TrN+G	7509.901	3656.951
ADK	TIM2+I+G	6268.198	3062.099
UCEH	HKY+G	1362.831	638.407

Notes: AIC: Akaike Information Criterion; −lnL: negative log likelihood.

**Figure 2:**
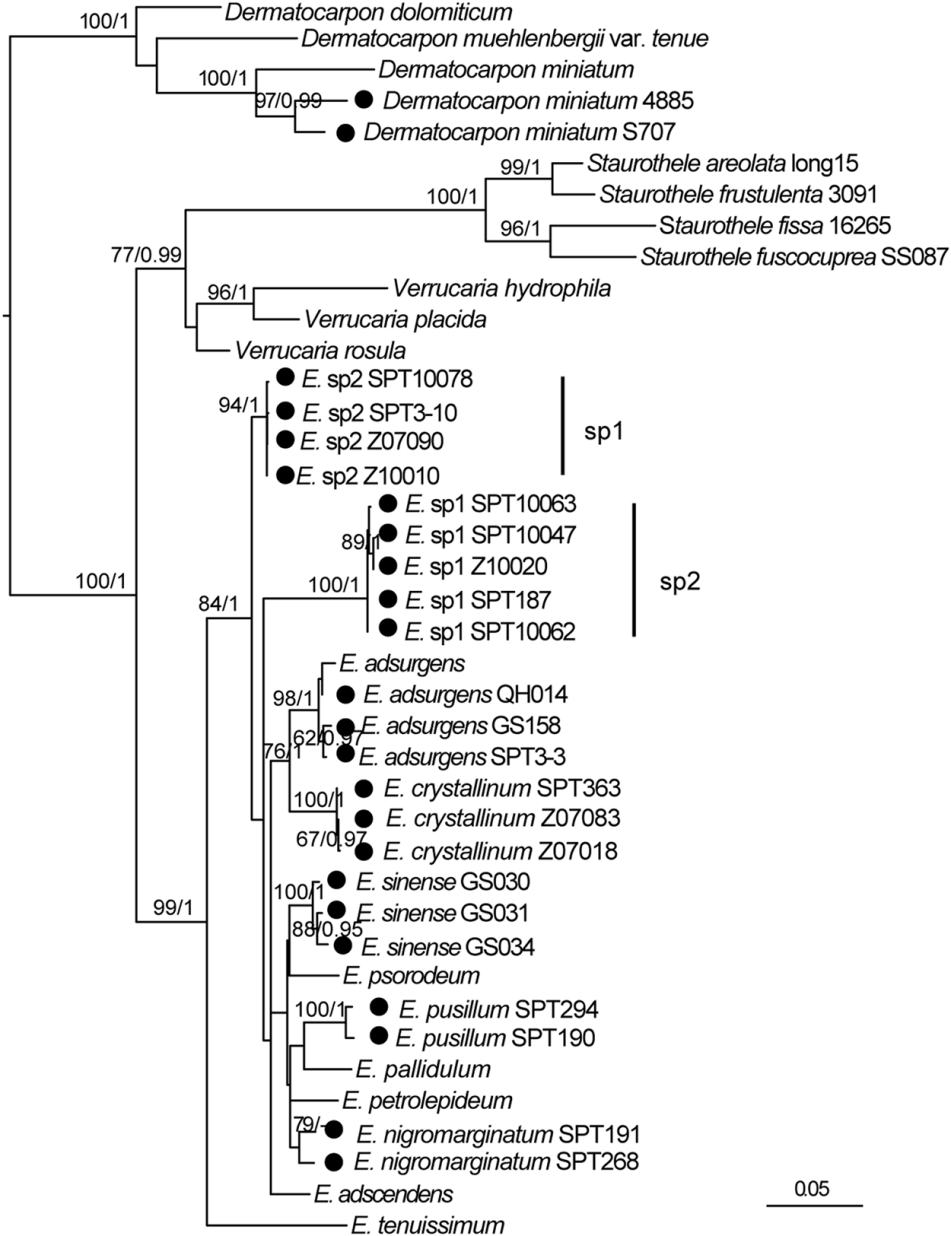
The maximum likelihood tree of *Endocarpon* species based on the concatenated ITS, ADK and UCEH sequences using the partition model. The numbers in each node represent bootstrap support (BS) and posterior probability (PP) values based on Bayesian analysis; numbers lower than 70 (BS) and 0.95 (PP) are not shown. Bootstrap values ≥75 and posterior probability values ≥95 were plotted on the branches of the RAxML tree. Newly generated sequences are marked with the symbol. combined with closely related sequences downloaded from GenBank. Scale = 0.05 substitution per site.

The concatenated three-locus data sets contained 40 sequences (1434 nucleotides sites), comprising 11 *Endocarpon* species. The maximum likelihood (ML) tree for the concatenated data sets constructed using partitioned models are presented in Fig. 2. The maximum likelihood analyses (ML, RAxML) shows the same highly supported clades as the Bayesian analyses. Both analyses were merged in one phylogenic tree, and the respective values (bootstrap values ≥75, posterior probability values ≥95) were plotted directly on the branches (Fig. 2). Based on the phylogenetic results the genus *Endocarpon* forms a strongly supported monophyletic clade and is obviously separated from the other genera within Verrucaricaceae, i.e., *Dermatocarpon* spp., *Staurothele* spp., *Verrucaria* spp. and *Willeya* spp. Within the *Endocarpon* clade, all 11 studied species formed highly supported lineages.

### Scanning electron microscope (SEM) images of rhizines in the two new species

**Figure 3:**
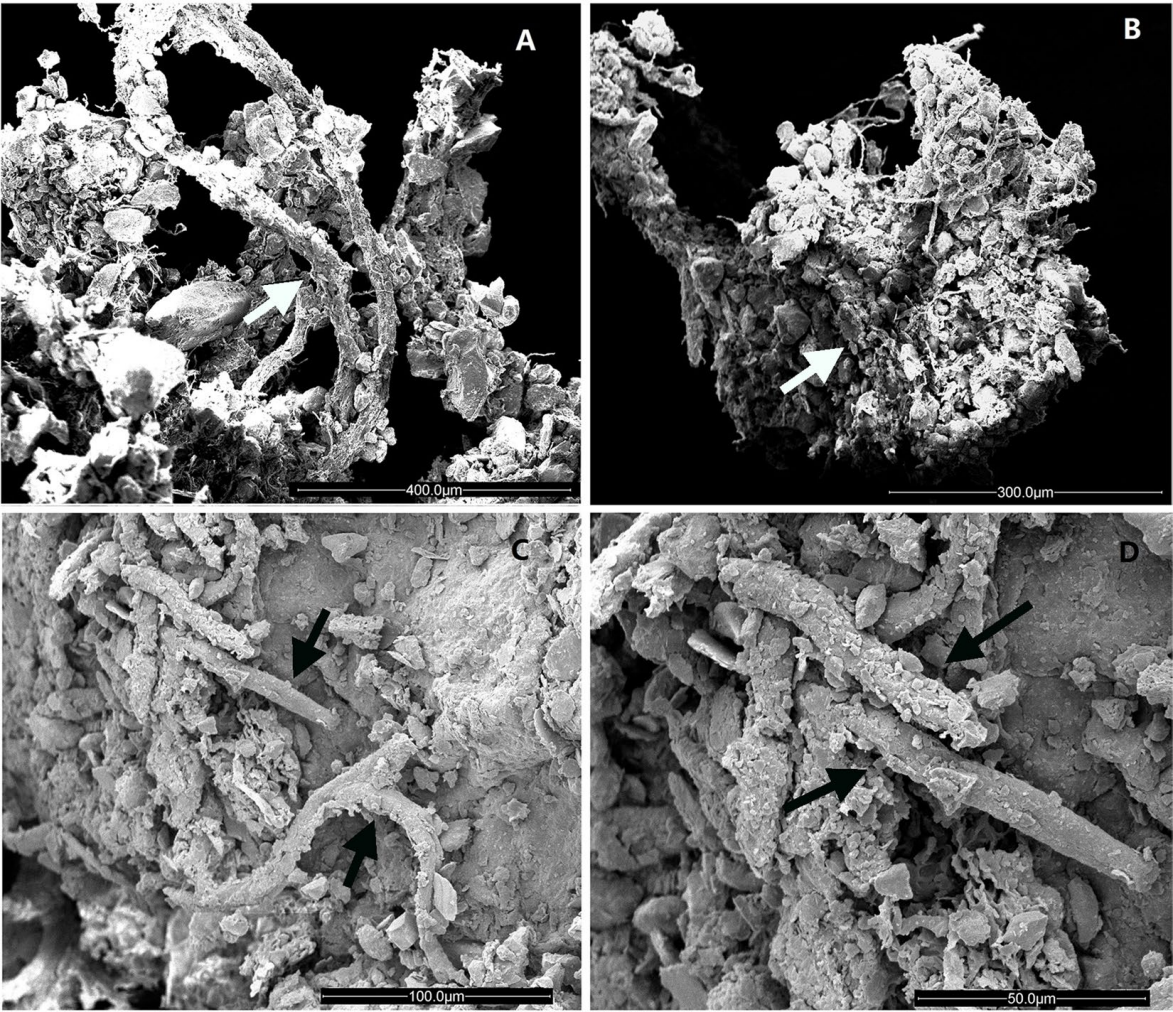
SEM images of thallus rhizines binding sand particles. (**A**,**B**) *Endocarpon deserticola* (holotype, Z07090); (**C**,**D**) *Endocarpon unifoliatum* (holotype, Z10020). Arrows pointing to the rhizines.

Both of the new *Endocarpon* species may fulfil potentially important roles by stabilising soils via sand particles consolidation with their rhizines, as inferred from SEM observations (Fig. 3). The sand particle surface is covered by the squamose thalli of the *Endocarpon* species (Fig. 1C), and sand crystals are wrapped in their branched rhizines (Fig. 3).

## Discussion

### Phenotype

**Figure 4:**
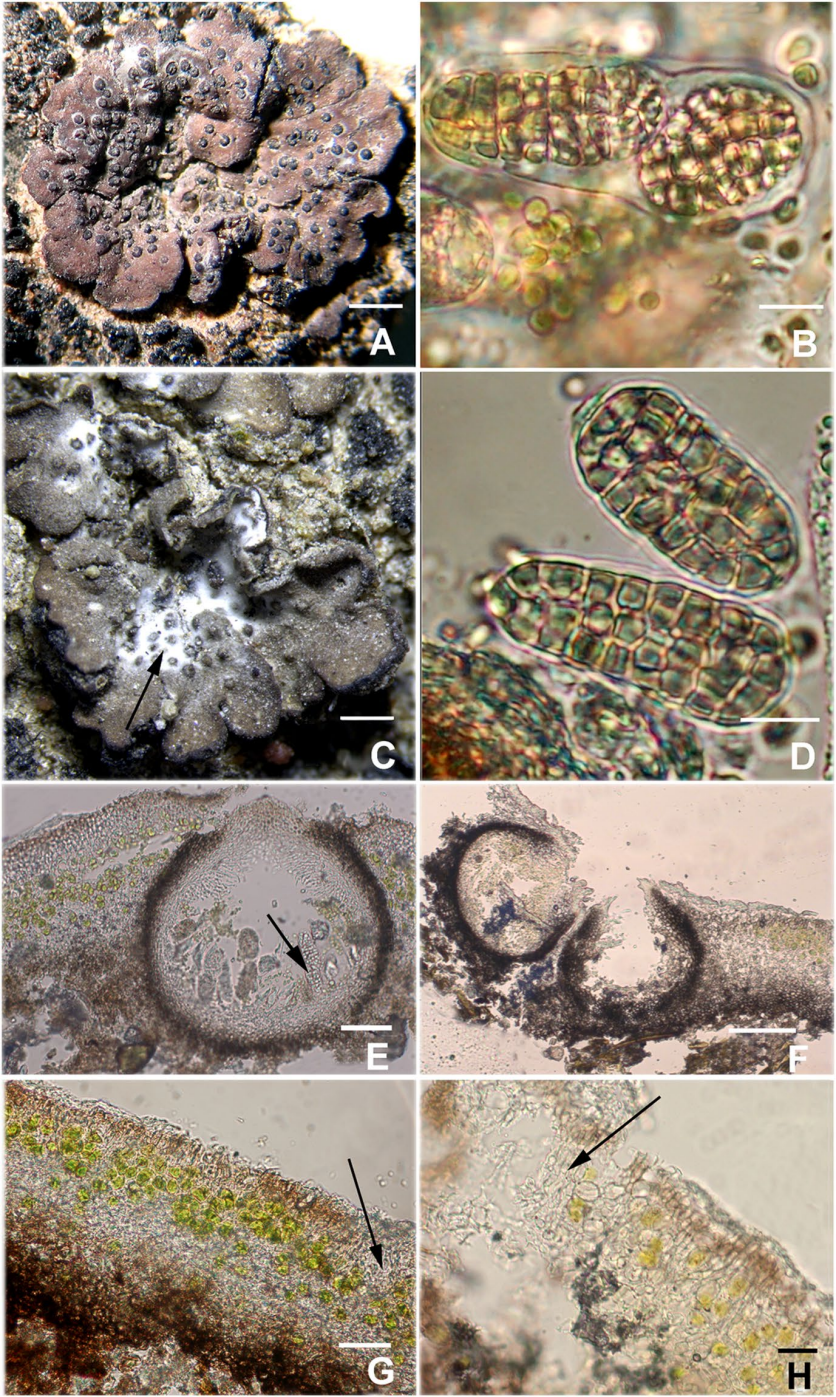
Endocarpon deserticola: (**A**) Upper surface of squamae with abundant perithecia (holotype, Z07090), scale bar = 1 mm; (**B**) an ascus containing two ascospores (paratype, SPT3–10), scale bar = 10 µm. *Endocarpon unifoliatum*: (**C**) upper surface of unifoliate squama with slightly upturned margins (holotype, Z10020), the arrow pointing to white portion of thallus, scale bar = 0.5 mm; (**D**) muriform ascospores (paratype, SPT10063), scale bar = 10 µm. (**E**) Anatomic structure of perithecia of *Endocarpon deserticola* (holotype, Z07090), the arrow pointing to ascospores, scale bar = 50 µm. (**F**) Anatomic structure of perithecia of *Endocarpon unifoliatum* (holotype, Z10020), scale bar = 100 µm; (G) Anatomic structure of thallus (holotype, Z10020), the arrow pointing to white part of upper cortex, scale bar = 50 µm. (**H**) Partial magnification of anatomic structure of thallus (holotype, Z10020), the arrow pointing to white portion of the upper cortex and indicating less to absence of algal cells in this part, scale bar = 20 µm.

According to our morphological assessment, some samples collected from the Tengger Desert in China were not able to be categorised as any previously described *Endocarpon* species^9, 15, 31–45^. Samples corresponding to the newly described *Endocarpon deserticola* are characterised by abundant perithecia dispersed throughout nearly all squamules, and the perithecia reach 15–60 (up to 100) in number (Fig. 4A). This species is most similar to *E. helmsianum* Müll. Arg. of Australia, which is also characterised by abundant perithecia^41^; however, *E. helmsianum* exhibits wider squamules (5–25 mm), a more contiguous to overlapping thallus, and much larger ascospores^41^.

Samples corresponding to *Endocarpon unifoliatum* are characterised by a unifoliate, concave, and lobated thallus with slightly upturned margins (Fig. 4C). This species is similar to *E. pusillum* Hedw., but distinguished by plane, fully adnate thallus and tightly aggregated, nearly inseparable squamules in *E. pusillum* ^42^.

### Molecular phylogeny

The genus *Dermatocarpon* with foliose umbilicate thallus was strongly supported (BS = 100%, PP = 1) separating from other genera with crustose or squamulose thallus (*Endocarpon*, *Staurothele* and *Verrucaria*). Another genus with hymenial algae besides *Endocarpon*, *Staurothele*, clustered closer to *Verrucaria* (BS = 77%, PP = 0.99) than to *Endocarpon*.

Within the *Endocarpon* clade was no explicit interspecific relationship reflected in the phylogenetic tree (Fig. 2), which may be the results of the limited number of species and gene loci included in the analyses. Nevertheless, each species was highly supported and obviously separated from others, e.g. the two new putative species *Endocarpon deserticola* and *Endocarpon unifoliatum*, formed two strongly supported clades separated from other species. The two newly developed protein coding markers (ADK and UCEH) show a quite high phylogenetic informative signal on species level and can be useful for future phylogenetic analyses, not only for *Endocarpon*, but also for other fungal genera (Figures S1–S3). This finding confirms the reliability of gene trees for phylogenetic analyses based on concatenated data sets.

The species *Endocarpon tenuissimum* is nested within the monophyletic *Willeya diffractella* (Nyl.) Müll. Arg. and has been placed in synonymy^30^, which is also supported by the ML tree based on ITS sequences in the present study (Figure S1). However, within the monophyletic *Willeya diffractella* clade, neither morphology nor geography was found to be corresponding to the main infraspecific groupings except the nature of the substrate (calcareous vs non-calcareous). As known *Endocarpon tenuissimum* shared the same character, i.e. non-calcareous substrate, with one group of *Willeya diffractella*, but there are some subtle differences in thallus color, ecology and ascospore size between *Endocarpon tenuissimum* and *Willeya diffractella* ^30^. More samples and genes are required to further explore the consistency between phenotype and phylogeny within *Willeya diffractella*.

#### Role of rhizines in soil stabilisation

Both new species of *Endocarpon* may fulfil important roles by stabilising soils by sand particle consolidation with their rhizines, which were inferred from SEM observations (Fig. 3). This finding is consistent with previous studies investigating lichenised BSC fungal communities in desert ecosystems^15, 51^. Lichens are more effective than cyanobacteria at reducing soil erosion because the fungal hyphae of the lichen thallus penetrate more deeply and the tissue extends above the soil surface^52^.

In summary, based on morphological and molecular phylogenetic data, two new putative species, *Endocarpon* sp. 1 and *Endocarpon* sp. 2, have been described under the names *Endocarpon deserticola* and *Endocarpon unifoliatum*, respectively. In previous studies, the lichen species *Endocarpon pusillum* exhibited drought resistance^15^ and sand and carbon fixation^9, 11^, and thus served as an important species for desert bio‐carpet engineering and the study of stress tolerance mechanisms in lichens in China^12–14^. The two new species, *E. deserticola* and *E. unifoliatum*, exhibit different morphological and phylogenetic characteristics from those of *E. pusillum* and may also play important roles in desert sand stabilisation. Further characterisation of features such as stress tolerance and carbon fixation should be carried out in the near future. Additionally, genomic studies should be performed to learn more about the mechanisms of stress tolerance in lichens.

### Taxonomy

***Endocarpon deserticola*** T. Zhang, X. L. Wei & J. C. Wei, sp. nov., Fig. 4A,B

Fungal Name: FN570287

TYPUS: China, Ningxia Hui Autonomous Region, Zhongwei city, Shapotou north experimental zone, on soil crust, January 2007, Zhangtao, Z07090 (holotypus-HMAS-L-135062).

Etymology: The epithet of the new species ‘*deserticola*’ is a Latin compound consisting of the Latin noun ‘*desertum*: *desert*’ and the Latin adjective suffix ‘-*colus*: *inhabiting*’, meaning that the new species grows in the desert.

Diagnosis: This species is characterised by its abundant perithecia, which is dispersed throughout almost all squamules, up to 100 or more in number.

Morphology: Thallus terricolous, squamulose; squamules solitary or contiguous, with slightly upturned margins, rounded, elongate or irregular, 1–3 (−4) mm in width; upper surface pale brownish to brownish; lower cortex well-developed, dark brown to black, with brown to black rhizines, 4–6 mm long, irregularly branching in the terminal region.

Upper cortex 19.5–27 (−37.5) µm thick, consisting of three layers: amorphous layer 15–22.5 (−30) µm thick; middle layer pale brown, 7.5–15 (−18) µm thick, paraplectenchymatous; lower layer hyaline, 22.5–37.5 µm thick; algal layer 45–60 (−67.5) µm thick, consisting of coccoid green microalgae, algal cells globose (diameter 3–6 µm) or subglobose (4.5–7.5 × 3–4.5 (−6) µm) and bright green; medulla pale whitish, 15–45 (−60) µm thick, without sharp demarcation between it and algal layer; lower cortex (from 22.5 to) 30–45 µm thick, dark brown to black.

Ascomata perithecioid, subglobose, immersed in thallus, 15–60 (−100) per squamule, brown to dark brown. Perithecia obpyriform, 200–275 (−325) × 200–250 µm; excipulum dark brown, 30–45 µm thick at the base and sides, pale brown at the apex near the ostiole; periphyses mostly simple, 22.5–37.5 (−45) µm long; hymenial algal cells globose (diameter 3–4.5 µm) to subglobose (3–4.5 × 1.5–3 µm), green; asci bisporous, clavate, 67.5–75 × 15–24 µm; ascospores muriform, two per ascus, hyaline to slightly brownish, matured spores brown, elongate-ellipsoid to subcylindrical, with 2–5 (−6) transverse divisions and 6–12 (−15) longitudinal divisions; upper spore generally broader and shorter than lower spore; upper spore: 28.5–39 × 18–22.5 µm; lower spore (from 31.5 to) 37.5–45 × 13.5–18 µm.

Pycnidia not seen.

Chemistry: K-, C-, KC-, P-; no lichen substances detected using thin-layer chromatography (TLC).

Substrate: Calcareous sands.

Additional specimens examined: China, Ningxia Autonomous region: Zhongwei city: Shapotou north experimental zone: soil crust, April 8, 2010, T. Zhang, Z10010 (HMAS-L-134712); soil crust, August 31, 2010, T. Zhang, SPT3–10 (HMAS-L-134716); soil crust, April 10, 2010, T. Zhang, SPT10078 (HMAS-L-121580); soil crust, April 18, 2007, J. Yang & T. Zhang, SPT295 (HMAS-L-134714); soil crust, August 6, 2003, J. C. Wei, SPT005 (HMAS-L-134713).

Comments: This species is most similar to *E. helmsianum* found in Australia, which is also characterised by abundant perithecia. However, *E. helmsianum* has wider squamules (5–25 mm), a more contiguous-to-overlapping thallus, and much larger ascospores.

***Endocarpon unifoliatum*** T. Zhang, X. L. Wei & J. C. Wei, sp. nov., Fig. 4C–F

Fungal Name: FN570274

TYPUS**:** China, Ningxia Hui Autonomous Region, Zhongwei city, Shapotou north experimental zone, on soil crust, April 8, 2010, T. Zhang, Z10020 (holotypus-HMAS-L-134711).

Etymology: The epithet of the new species ‘*unifoliatum*’ is the nominative singular neuter of the Latin adjective ‘*unifoliatus*: *with one thallus’*.

Diagnosis: This species is characterised by its unifoliate, concave, lobate thallus with slightly upturned margins.

Morphology: Thallus terricolous, squamulose, concave, and lobate, sometimes greyish-white to white at the central part of thallus, brown at the thallus edges; squamules mostly solitary, not contiguous, with slightly upturned margins, rounded, elongate or irregular, 1–2 (−4) mm wide; upper surface pale to yellowish brown; lower cortex well developed, dark brown to black, with black rhizines 2–3 mm long, irregularly branching in the terminal region.

Upper cortex 19.5–27 (−37.5) µm thick, consisting of three layers: amorphous layer 1.5–3 µm or absent; middle layer dark brown, 12–15 µm thick, paraplectenchymatous; the lower layer hyaline, 7.5–12 (−22.5) µm thick. Algal layer 30–45 (−52.5) µm thick, consisting of coccoid green microalgae, algal cells globose (diameter 6–7.5 µm) or subglobose (6–7.5 × 3–4.5 (−6) µm) and bright green. Medulla pale whitish, 15–37.5 µm thick, merging indistinctly to the algal layer. Lower cortex 7.5–15 (−18) µm thick, dark brown to black.

Ascomata perithecioid, subglobose, immersed in thallus, 0–10 (−20) per squamule, brown to dark brown. Perithecia obpyriform, 225–275 (−300) × 175–250 µm; excipulum dark brown, 20–37.5 µm thick at the base and sides, pale brown at the apex near the ostiole; periphyses mostly simple, 22.5–34.5 µm long; hymenial algal cells globose (diameter 2–3 µm) to subglobose (3 × 1.5 µm), green; asci bisporous, clavate, 51–67.5 (−75) × 18–19.5 µm; ascospores muriform, two per ascus, hyaline to slightly brownish, matured spores brown, elongate-ellipsoid to subcylindrical, with 2–4 (−5) transverse divisions and 6–9 longitudinal divisions, upper spore generally broader and shorter than lower spore; upper spore 22.5–30 (−37.5) × 13.5–18 µm; lower spore 27–34.5 (−37.5) × 12–15 µm.

Pycnidia not seen.

Chemistry: K-, C-, KC-, P-; no lichen substances detected by TLC.

Substrate: Calcareous sands.

Additional specimens examined: China, Ningxia Autonomous region: Zhongwei city: Shapotou north experimental zone: soil crust, April 9, 2010, T. Zhang, SPT10062 (HMAS-L-134709); soil crust, April 9, 2010, T. Zhang, SPT10063 (HMAS-L-121315); soil crust, April 9, 2010, T. Zhang, SPT10047 (HMAS-L-134668); soil crust, April 19, 2007, J. Yang & T. Zhang, SPT187 (HMAS-L-134708).

Comments: This species is similar to *E. pusillum*, but *E. pusillum* is delimited by its plane, fully adnate thallus, and tightly aggregated, nearly inseparable squamules.

## Materials and Methods

### Lichen collection and ethics statement

Lichen specimens were collected from the Shapotou region (37°32′N, 105°02′E) on the southeast fringe of the Tengger Desert (Fig. 1). The investigation areas are located at an elevation of 1339 m in the steppified desert zone, which is also a transitional zone between desert and oasis^53^. The area has a mean annual precipitation of 180.2 mm, a mean annual evaporation of 3000 mm, a mean annual air temperature of 10.0 °C (minimum −25.1 °C, maximum 38.1 °C), an annual sunshine duration of 3264 h, a mean annual wind velocity of 2.9 ms^−1^, and 59 annual dust-storm days^54^. Ethical approval for lichen collection was obtained from the Shapotou Desert Research and Experimental Station. All species were deposited in the Herbarium Mycologicum Academiae Sinicae - Lichenes (HMAS-L).

### Morphological and anatomical analyses

A dissecting microscope (ZEISS Stemi SV11) and compound microscope (ZEISS Axioskop 2 plus) were used to study the phenotypic traits of the specimen. Colour test reagents (10% aqueous KOH, saturated aqueous Ca(OCl)_2_, and concentrated alcoholic p-phenylenediamine) and TLC (solvent system C) were used to detect lichen substances^55, 56^.

### DNA extraction, PCR amplification, and sequencing

Thirty-six specimens, including seven *Endocarpon* species, were chosen for DNA extraction, as shown in Table 1. The extraction procedure followed the modified CTAB method^57^. Three gene loci were used for PCR amplification: the nrDNA ITS region and two protein-coding genes, ADK and UCEH. The primer pairs ITS4 and ITS5^58^ were used to amplify the nrITS regions, and the primers for ADK and UCEH were newly designed in this study (Table 3) based on the cDNA library of *E. pusillum* ^50^. The PCR reaction was carried out as follows: pre-denaturation at 95 °C for 8 min, followed by 35 cycles of amplification [95 °C for 50 s, 53 °C (50 °C for UCEH) for 50 s, 72 °C for 1 min], and finally followed by extension for 8 min at 72 °C. The PCR products were purified and sequenced by Genewiz Inc., Beijing, China.

**Table 3:** Primers used for PCR amplification in this study.

Primer	Gene loci	Sequence (5′ → 3′)	Reference
ITS4	ITS	GGAAGTAAAAGTCGTAACAAGG	White *et al*.^44^
ITS5	ITS	TCCTCCGCTTATTGATATGC	White *et al*.^44^
325 F	UCEH	GATGTCATCAACCAAACCTG	This study
325 R	UCEH	TCATACATCCTCCATCGC	This study
ADK1	ADK	ATGGCGCCAATTASGGATGACACGGTCACCGACCTGAAGGAT	This study
ADK2	ADK	CAGTCCAATCTTGCTCAGAATGCTGCTCCC	This study

### Phylogenetic analyses

The sequences generated for this study were complemented with sequences from GenBank representing additional specimens or species, as listed in Table 1. The gene sequences of three loci, specifically nrDNA ITS, ADK and UCEH, were used for phylogenetic analyses. Sequences were aligned using ClustalW Multiple Alignment^59^ in BioEdit 7.2.5^60^ and introns were manual excluded. The alignment files were transformed into both phylip and nexus formats using SeaView version 4^61, 62^. The best model for the three single genes used in the phylogenetic analysis was identified in advance with jModelTest-2.1.9^63, 64^.

#### Congruence among loci

To test the level of congruence among loci, highly supported clades (equal to or more than 75% bootstrap) from single-gene trees were compared and assessed^65, 66^. Each locus was subjected to a randomised accelerated maximum likelihood (RAxML) analysis involving 1000 pseudoreplicates with RAxML-HPC BlackBox 8.2.6 (Stamatakis 2014) on the Cipres Science Gateway (http://www.phylo.org)^67^. The results were visualised with FigTree 1.4.2. When there was no conflict using a 75% bootstrap value threshold, in situations where a monophyletic group was supported with bootstrap values ≥75% at one locus and the same group of taxa was supported (≤75%) as non-monophyletic with another locus, the group was assumed to be congruent and the data set was concatenated^66^.

#### Phylogeny of the genus *Endocarpon*

Phylogenetic analyses of *Endocarpon* were performed using the concatenated data set, which was analysed using RAxML-HPC BlackBox 8.2.6^68^ and MrBayes 3.2.6^69, 70^ on the Cipres Science Gateway (http://www.phylo.org)^67^. For the ML analysis, the GTR+G+I model was used as the substitution model with 1000 pseudoreplicates. The data were partitioned according to the different genes. The best model for the three single genes used in the Bayesian analysis was obtained in advance with jModelTest-2.1.9. Data sets for the two protein-coding genes (ADK and UCEH) were also partitioned by codon position. Two parallel Markov chain Monte Carlo runs were performed, each using 8000000 generations and sampling every 1000 steps. A 50% majority rule consensus tree was generated from the combined sampled trees of both runs after discarding the first 25% as burn-in.

### Scanning electron microscopy

Rhizines of the samples were observed by performing SEM. Samples were sputter-coated with gold particles using a Bio-Rad SEM coating system (Sputter Coater BALTEC SDC 005, Leica Microsystems, Liechtenstein), and SEM images were recorded using a scanning electron microscope (SEM Quanta-200, FEI, Czech Republic) with a secondary electron detector operated at 10.0 kV.

### Nomenclature

The electronic version of this article in Portable Document Format (PDF) in a work with an ISSN or ISBN will represent a published work according to the International Code of Nomenclature for algae, fungi, and plants. In addition, new names contained in this study have been submitted to Fungal Names (FN) from where they will be made available to the Global Names Index. The unique FN number can be resolved and the associated information viewed through any standard web browser by appending the FN number contained in this publication to the prefix http://www.mycobank.org/MB/.

## Electronic supplementary material

Figure S1 to S3
