# Supplementary material for: Two new species of Endocarpon (Verrucariaceae, Ascomycota) from China
Source: Sci Rep. 2017 Aug 3;7:7193. doi: 10.1038/s41598-017-07778-5 (PMC5543127; doi:10.1038/s41598-017-07778-5)
Supplement: Supplementary file 1 — Figure S1 to S3 [file 41598_2017_7778_MOESM1_ESM.pdf]

# Two new species of *Endocarpon* (Verrucariaceae, Ascomycota) from China

Tao Zhang[1], Meng Liu[1], Yan-Yan Wang[1], Zhi-Jun Wang[1,2], Xin-Li Wei[1]*, Jiang-Chun Wei[1,3]*

[1] *State Key Laboratory of Mycology, Institute of Microbiology, Chinese Academy of Sciences, Beijing 100101, PR China*

[2] *The College of Life Science, Southwest Forestry University, Kunming 650224, PR China*

[3] *University of Chinese Academy of Sciences, Beijing 100049, PR China*

*Co-corresponding authors: Xin-Li Wei, weixl@im.ac.cn; Jiang-Chun Wei, weijc2004@126.com*

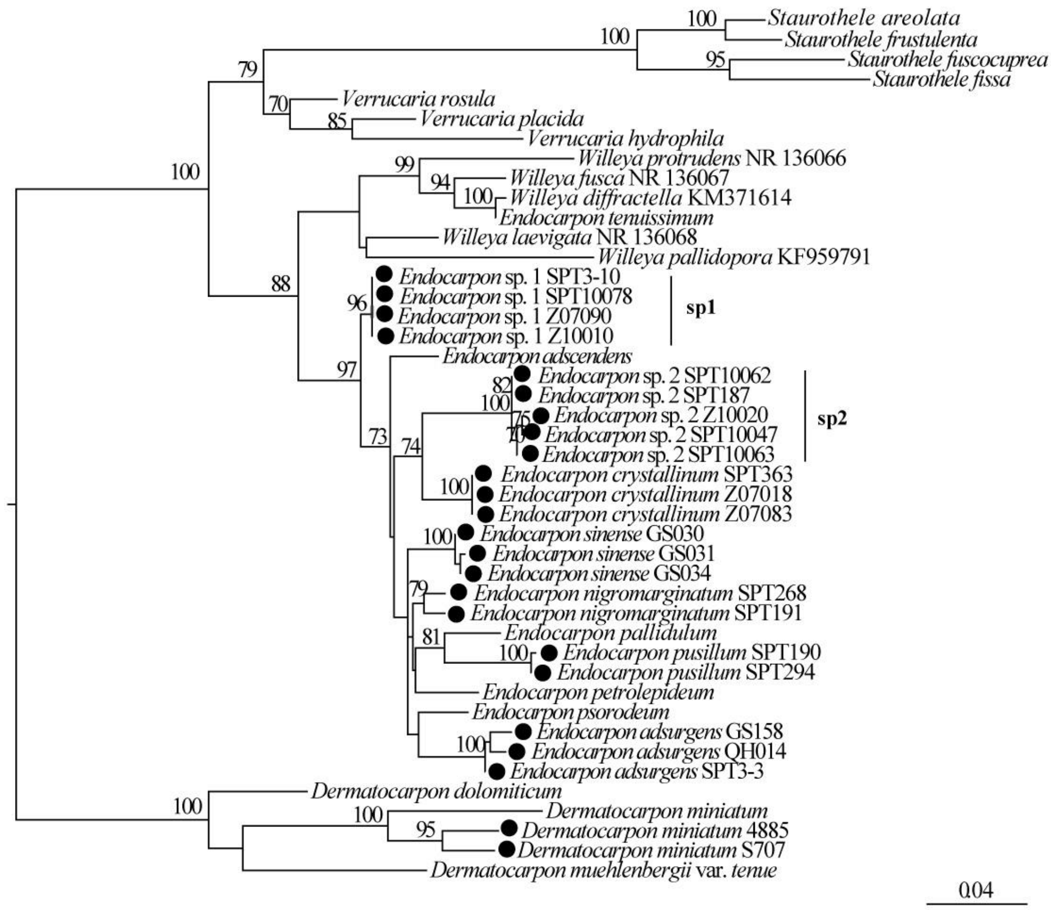


**Figure S1| The maximum likelihood tree of *Endocarpon* species based on ITS sequences.** The numbers in each node represents bootstrap support value, and the numbers lower than 70 were not shown. The samples marked by the symbol ● indicate that their DNA sequences are newly generated in this study, and others are based on the sliced DNA sequences in each species downloaded from GenBank. Scale=0.04 substitution per site.

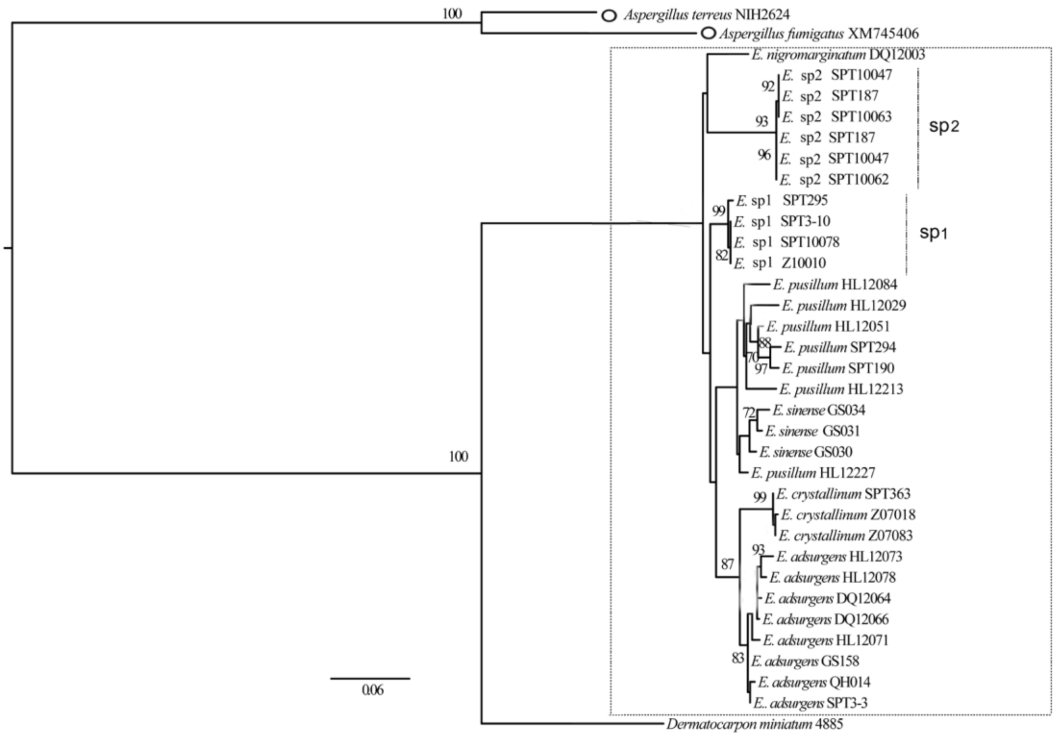


**Figure S2| The maximum likelihood tree of *Endocarpon* species based on ADK sequences.** The numbers in each node represents bootstrap support value, and the numbers lower than 70 were not shown. The samples marked by the symbol ○ were from GenBank, and other sequences were newly generated in this study. The ingroup of all the *Endocarpon* species are framed by the outermost broken-line box, within which the two new species are pointed out by the dash and broken-line boxes. Scale=0.06 substitution per site.

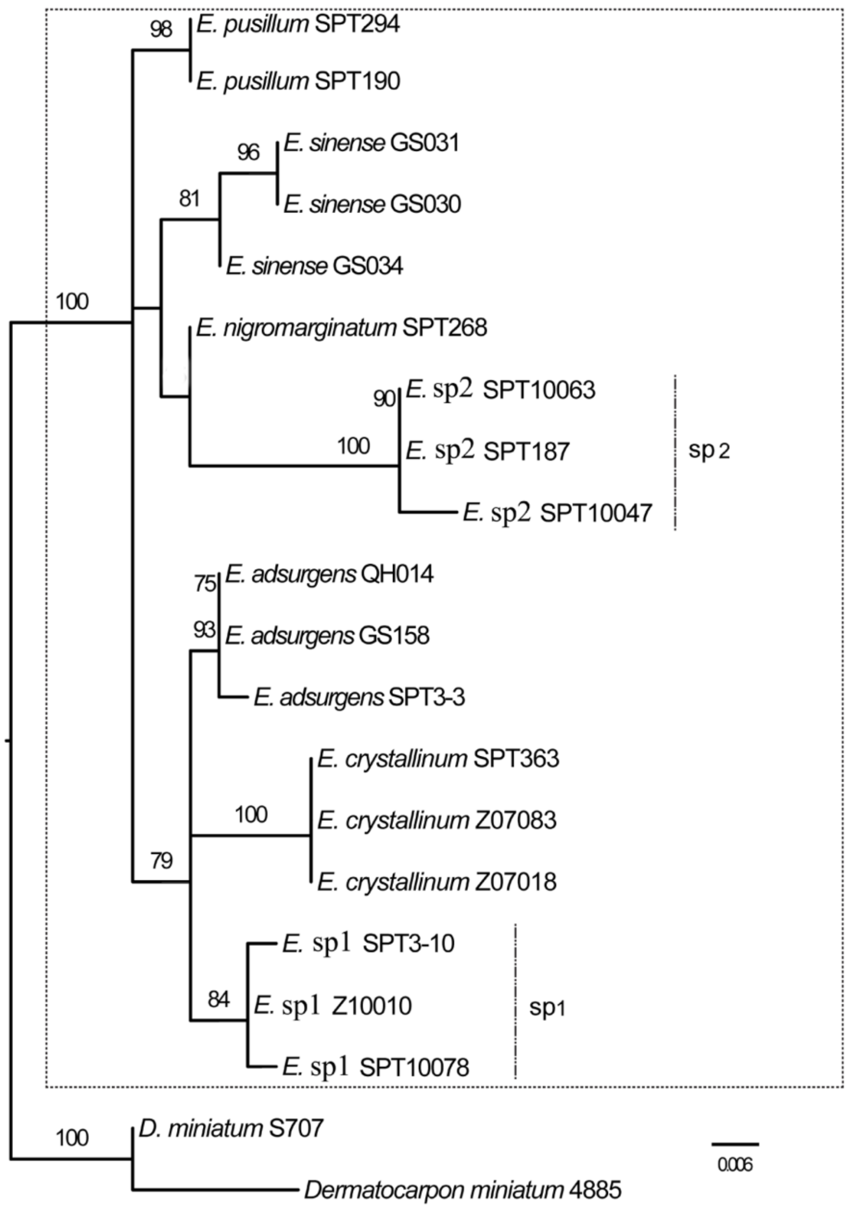


**Figure S3| The maximum likelihood tree of *Endocarpon* species based on UCEH sequences.** The numbers in each node represents bootstrap support value, and the numbers lower than 70 were not shown. All the sequences were newly generated in this study. The ingroup of all the *Endocarpon* species are framed by the outermost broken-line box, within which the two new species are pointed out by the dash and broken-line boxes. Scale=0.006 substitution per site.
